# Supplementary material for: Puerarin Alleviates H2O2-Induced Oxidative Stress and Blood–Milk Barrier Impairment in Dairy Cows
Source: Int J Mol Sci. 2023 Apr 24;24(9):7742. doi: 10.3390/ijms24097742 (PMC10178507; doi:10.3390/ijms24097742)
Supplement: Supplementary file 1 [file ijms-24-07742-s001.zip › ijms-2362357-supplementary.pdf]

Table S1. Milk yield and components of lactating dairy cows (n = 20) after PUE supplementation in the diet for 7 days.

	Time	Control	Control + PUE	Mastitis	Mastitis + PUE
Milk yield (kg	0d	29.30 ± 1.41	29.62 ± 0.97	19.95 ± 2.70 [b]	20.65 ± 3.15 [b]
per milking)	7d	29.72 ± 1.78	30.63 ± 1.13	17.79 ± 3.08 [b]	22.45 ± 3.27 [abc]
Protein, %	0d	3.41 ± 0.10	3.39 ± 0.08	2.95 ± 0.06 [b]	3.00 ± 0.08 [b]
	7d	3.47 ± 0.07	3.54 ± 0.10 [a]	2.86 ± 0.06 [b]	3.11 ± 0.08 [abc]
Fat, %	0d	4.29 ± 0.10	4.16 ± 0.11	3.29 ± 0.11 [b]	3.28 ± 0.14 [b]
	7d	4.31 ± 0.13	4.28 ± 0.13	3.19 ± 0.11 [ab]	3.41 ± 0.11 [abc]
Lactose, %	0d	5.01 ± 0.10	5.04 ± 0.09	4.43 ± 0.17 [b]	4.52 ± 0.25 [b]
	7d	5.13 ± 0.10	5.18 ± 0.07	4.37 ± 0.14 [b]	4.63 ± 0.26 [abc]
Other	0d	5.87 ± 0.07	5.80 ± 0.04	5.52 ± 0.17 [b]	5.55 ± 0.19 [b]
solids, %	7d	5.90 ± 0.09	5.86 ± 0.03	5.45 ± 0.15 [b]	5.66 ± 0.20 [b]
SCC× 1，000	0d	72.35 ± 12.19	83.90 ± 9.22	321.09 ± 71.45 [b]	308.00 ± 74.24 [b]
	7d	74.65 ± 12.28	81.20 ± 9.38	346.94 ± 73.09 [ab]	272.74 ± 76.59 [abc]

[a] indicates a significant difference in the corresponding on day 0 and day 7 in the same group ($P < 0.05$), [b] indicates a significant difference relative to the control group at the same time ($P < 0.05$), and [c] indicates a significant difference with the mastitis group at the same time ($P < 0.05$). All experiments were repeated three times, and experiments were performed simultaneously using different individual cows. The data are shown as the mean ± SD. Allow comparisons between the two groups were performed by unpaired two-tailed Student’s t test.

Table S2. Primers used for RT-qPCR.

Primers name	Sequence (5′-3′)
β-actin F	CCCTGGAGAAGAGCTACGAG
β-actin R	GTAGTTTCGTGAATGCCGCAG
IL-6 F	GCTGAATCTTCCAAAAATGGAGG
IL-6 R	GCTTCAGGATCTGGATCAGTG
IL-8 F	AAGCTGGCTGTTGCTCTCTT
IL-8 R	GGTGGAAAGGTGTGGAATGT
CCL5 F	GCCTTGAACCTGAACTTGCG
CCL5 R	TGGAATCTGTGCCTTCCCAG
BAX F	TGCTTCAGGGTTTCATCC
BAX R	CTTCAGACACTCGCTCAG

BCL-2 F	TTCTCCTGGCTGTCTCTG
BCL-2 R	CTGCTTCTTGAATCTTCTGC
Claudin-4 F	ACGTCATCCGCGACTTCTAC
Claudin-4 R	ACTTAGCGGAGTAGGGCTTG
Occludin F	GCTTGTTATCGTGATGTGC
Occludin R	TGGGATCTGTATAGCCTGTT
ZO-1 F	CCTGCCCAACTCAACTCAT
ZO-1 R	TGGTCACTTCTTCTCCTTTT
Symplekin F	GCAGCGTGCGTAAGAATC
Symplekin R	GCTCCAAGTCCTTGTCCTC
Nrf2 F	CCAGCACAACACATACCATCAG
Nrf2 R	CGTAGCCGAAGAAACCTCATTG
HO-1 F	GGCAGCAAGGTGCAAGA
HO-1 R	GAAGGAAGCCAGCCAAGAG
xCT F	GATACAAACGCCCAGATATGC
xCT R	ATGATGAAGCCAATCCCTGTA
